# Gender and age detection assist convolutional neural networks in classification of thorax diseases

**DOI:** 10.7717/peerj-cs.738

**Published:** 2021-11-09

**Authors:** Mumtaz Ali, Riaz Ali

**Affiliations:** 1 Computer Science, Huazhong University of Science and Technology, Wuhan, Hubei, China; 2 Computer Systems Engineering, Sukkur IBA University, Sukkur, Sukkur, Sindh, Pakistan; 3 Computer Sceince, Sukkur IBA University, Sukkur, Sukkur, Sindh, Pakistan

**Keywords:** Gender, Age, Thorax disease classification, Convolutional neural networks

## Abstract

Conventionally, convolutional neural networks (CNNs) have been used to identify and detect thorax diseases on chest x-ray images. To identify thorax diseases, CNNs typically learn two types of information: disease-specific features and generic anatomical features. CNNs focus on disease-specific features while ignoring the rest of the anatomical features during their operation. There is no evidence that generic anatomical features improve or worsen the performance of convolutional neural networks for thorax disease classification in the current research. As a consequence, the relevance of general anatomical features in boosting the performance of CNNs for thorax disease classification is investigated in this study. We employ a dual-stream CNN model to learn anatomical features before training the model for thorax disease classification. The dual-stream technique is used to compel the model to learn structural information because initial layers of CNNs often learn features of edges and boundaries. As a result, a dual-stream model with minimal layers learns structural and anatomical features as a priority. To make the technique more comprehensive, we first train the model to identify gender and age and then classify thorax diseases using the information acquired. Only when the model learns the anatomical features can it detect gender and age. We also use Non-negative Matrix Factorization (NMF) and Contrast Limited Adaptive Histogram Equalization (CLAHE) to pre-process the training data, which suppresses disease-related information while amplifying general anatomical features, allowing the model to acquire anatomical features considerably faster. Finally, the model that was earlier trained for gender and age detection is retrained for thorax disease classification using original data. The proposed technique increases the performance of convolutional neural networks for thorax disease classification, as per experiments on the Chest X-ray14 dataset. We can also see the significant parts of the image that contribute more for gender, age, and a certain thorax disease by visualizing the features. The proposed study achieves two goals: first, it produces novel gender and age identification results on chest X-ray images that may be used in biometrics, forensics, and anthropology, and second, it highlights the importance of general anatomical features in thorax disease classification. In comparison to state-of-the-art results, the proposed work also produces competitive results.

## Introduction

Deep learning approaches based on convolutional neural networks (CNNs) have been widely used to classify thorax diseases in recent years. Almost all of these approaches use a pre-trained model such as AlexNet (Krizhevsky, Sutskever & Hinton, 2017), GoogleNet (Szegedy et al., 2015), ResNet (He et al., 2016), and DenseNet (Wang et al., 2017; Imane Allaouzi, 2019; Abiyev & Ma’aitah, 2018; Huang et al., 2017) or train CNNs from scratch. Since thorax diseases are only seen in a small portion of a chest X-ray, CNNs learn either disease-related features or outlier anatomical features. Traditionally, CNNs for thorax disease classification have been trained in such a way that they focus more on disease-related features while ignoring a lot of generic anatomical features. That approach is very conventional: what if we reverse the approach by training the model in such a way that it learns first the anatomical features, and then it learns the disease-related features? In that case, we may explore new insights into thorax disease classification. The generic anatomical features may play a vital role in thorax disease classification. Currently (up to our knowledge), there is no evidence in the literature that investigates the role of generic anatomical features to improve the performance of CNNs for thorax disease classification. Hence, if we train a model which learns generic anatomical features first and then uses that knowledge for thorax disease classification, there is a high chance of improving the performance of CNNs. To do so, we must first extract generic anatomical features. There can be at least three factors to obtain generic anatomical features: the model is designed in a specific way, the nature of the application for which the designed model is being trained, and the data for training is pre-processed in a certain way. Therefore, in this paper these three goals are accomplished in the following manner:

• As this is a well-established fact that initial layers of CNNs learn the edge and/or boundary-like features (Véstias, 2021), therefore we design a simple dual-stream CNN model. As each stream in the dual-stream model in our case starts with different kernel sizes it learns different edge and boundary-like features, and finally we combine those features for classification.
• We train the model initially for gender and age detection. This helps the model to focus solely on anatomical features.
• We pre-process the training data with Non-negative Matrix Factorization(NMF) (Gillis, 2014) and Contrast Limited Adaptive Histogram Equalization(CLAHE) (Zuiderveld & Heckbert, 1994) to enhance the edges and boundaries in the image which are predominantly related to anatomical features (Xue, Rajaraman & R, 2018).

The tasks mentioned previously are performed before the model is trained for thorax disease classification. The objective of the above-mentioned tasks (especially the gender/age detection and pre-processing of training data) is not just limited to improve the performance of CNNs for thorax disease classification, whereas they also present new insights. The gender and age detection task for instance presents new results that can be used in biometrics, forensics or anthropology, etc. Similarly, the pre-processing of training data with NMF and CLAHE can be helpful to enhance structural/anatomical features in similar applications. More precisely, after these tasks the model is trained in three phases; (1) The model is trained for gender detection on pre-processed data; (2) The model is trained again to detect age on pre-processed data; (3) The model is trained once again (this time on original data) to detect thorax diseases. To investigate the efficacy of our method we use the ChestX-ray14 dataset (Wang et al., 2017). The visualization investigation of obtained results is further scrutinized to conclude that the model learns features for gender and age detection from the portions of the image which are potential outliers for thorax disease classification. If the outlier features are already known the model may learn thorax diseases more perfectly.

### Our contributions

• Up to the best of our knowledge, this is the first work that investigates the role of anatomical features which are learned for gender and age detection to improve the performance of CNNs in thorax disease classification.
• We show that pre-processing chest X-ray images with NMF and CLAHE enhance the anatomical/structural features which later on help to improve the performance of CNNs for thorax disease classification.
• We highlight the important parts of the chest X-ray images for gender, age, and thorax disease classification using visualization analysis. Similarly, we show that the model learns disease-related features more effectively when anatomical features are already known.

## Related Work

Deep learning models have produced promising results for the problems based on chest X-ray images. Conventionally, the designing of a deep learning-based system for such problems consists of the stages of acquisition of the image/data, image/data pre-processing, extraction or selection of features, classification based on extracted features, and analysis of the performance. Feature extraction is one of the main stages of deep learning models because it takes an input image and transforms it into quantitative feature vectors. The methods for feature extraction for image processing in the literature are generally local phase quantization (LPQ), gray-level co-occurrence matrix (GLCM), threshold adjacency statistics, local binary pattern(LBP), parameter-free threshold adjacency statistics(PFTAS) (Vu et al., 2015). Similarly, local scale-invariant features, speeded up robust features, a bag of features, oriented FAST rotated BRIEF (ORB) key-point detector, discriminative feature-oriented dictionary learning method, and convolutional neural networks (CNNs) based feature extraction methods (Saxena, Shukla & Gyanchandani, 2020) are also very popular options.

Recently, feature extraction based on CNN has become a very effective method. The goals of this research are to improve the performance of CNNs in terms of anatomical features and offer new results for gender and age detection. To our knowledge, no research on the use of anatomical features to aid CNNs in learning features for thorax disease classification has yet been published. As a result, in this section, we’ll go through the literature review for detecting thorax diseases on chest X-ray images in connection with gender and age detection.

### Gender and age detection on chest X-ray images using CNNs

There is only scant evidence in the literature, which leverages deep learning methods for gender and age detection in the chest X-ray images. For instance, the authors (Xue et al., 2018) present a method to identify the gender of a person by using chest X-ray images. Their work is motivated to provide gender-specific labels to the un-annotated chest X-ray images. Their technique is based on four steps: pre-processing the X-ray images, feature extraction with CNNs, feature selection, and classification. They use pre-trained models, such as ResNet, AlexNet, GoogleNet, and VGGNet, for feature extraction. Then, they use Support Vector Machine (SVM) and Random Forest for feature selection. For the age detection, there are some notable contributions, especially, Domingues et al. (2016) use basic image processing methods to extract maximum width, and height of chest area within the chest X-ray image to predict the age of the subject. They build a computational framework based on logic programming for knowledge representation and reasoning.

### Thorax disease classification in chest X-ray images using deep learning

Disease detection in chest X-ray images is a challenging problem, researchers have adopted deep learning techniques to classify the chest X-ray images for chest diseases or thorax diseases. There are four main techniques to classify the chest X-ray images for thorax disease detection, namely dual-mode or two-stream networks, attention guided networks, squeeze-and-excitation blocks method, and traditional pre-trained models.

#### Dual-mode networks

In the dual-mode or two streams networks, the model is trained on the original data and pre-processed data, not only to learn features in a parallel fashion but also to achieve the feature concatenation. Teixeira et al. (2020) use a dual lesion attention network for thorax disease classification in chest X-rays images. Similarly, Liu et al. (2019) use a dual-mode of the DenseNet-121 network to classify the chest X-ray images for disease detection. Their model is based on two streams, where one stream extracts features from the overall input image, and the second stream extracts features from segmented lung regions in the image. Both streams are fused in the fully connected layer. Rubin et al. (2018) use a dual CNN model to detect thorax diseases on frontal view as well as lateral view chest X-ray images. There is a concatenation layer, which concatenates the outputs of the frontal view network and lateral view network.  Chen et al. (2019) use the frontal view chest X-ray images dataset to detect 14 thorax diseases. Their model is based on ResNet and DenseNet. They adopt a feature-level concatenation approach by concatenating features learned by both the streams of the model. The dual-mode networks perform well for disease detection in the chest X-ray image data, but they have a disadvantage of heavier computational cost.

#### Attention guided networks

The attention-guided networks are trained based on attention mechanism (Feng, Seong Teh & Cai, 2019). The localized attention improves the performance of the network by making a future reference point, hence the models focus more on a specific region, where the attention is pointing. Guan et al. (2020) present a method that uses attention guided technique to classify thorax diseases. They also present a multi-label chest X-ray image classification *via* category-wise residual attention learning (Guan & Huang, 2020).  Aurelia Bustos (2019) present a new dataset of 160,000 chest X-ray images, which are obtained from 67,000 patients. They use Recurrent Neural Networks (RNNs) with the attention mechanism to label the images.  (Guan & Huang (2018) also use a technique with an attention-guided mechanism to classify chest X-ray images for thorax disease detection. The attention-guided networks are not free from certain disadvantages and this type of network can easily be fooled (Feng, Seong Teh & Cai, 2019). There are several other drawbacks, *e.g.*, the model is dependent on the initial discovery of the features, thus some misleading features will influence the attention of the model.

#### Squeeze-and-excitation networks

In Squeeze-and-Excitation networks, the spatial and channel information of an image is fused. Akpinar, Genc & Karagol (2020) for instance use SqueezeNet (Iandola et al., 2016) to classify chest X-ray image as normal and abnormal. Similarly, Yan et al. (2018) propose a weakly supervised deep learning framework by leveraging squeeze-and-excitation blocks, multi-map transfer, and max–min pooling for classifying thorax diseases and localizing suspicious lesion regions. Ma et al. (2019) propose a multi-attention convolutional neural network for thorax disease classification and localization. Their framework uses a squeeze-and-excitation block as a feature attention module to combine global and local information. To alleviate the class imbalance problem, they present a hard-example attention module.

#### Pre-trained models

For these methods, the pre-trained models are fine-tuned to detect thorax diseases. Wang et al. (2017) present a new chest X-ray images dataset (denoted by chestX-ray8), which consists of 108,948 frontal view X-ray images of 32,717 patients with text mined labels for eight diseases. The authors evaluate four well-known models (AlexNet, GoogleNet, VGGNet, and ResNet-50) pre-trained on ImageNet. According to their performances, ResNet50 outperforms the other three models. Imane Allaouzi (2019) use DenseNet-121 architecture for multi-label classification to detect thorax diseases. Despite several advantages, such models lack their ability to avoid outlier features in a conventional single-mode configuration. The pre-trained models have a drawback of the inter-dependency of labels as well, therefore many authors have tried to overcome this problem. For instance, Yao et al. (2018) use LSTM network (Shi et al., 2015) instead of pre-trained models to classify 14 thorax diseases. They claim that their model ignores inter-dependencies among labels and outperforms the previous state-of-the-art models. Irvin et al. (2019) use DenseNet121 on a large chest X-ray images dataset named CheXpert. Their dataset contains 224,316 chest X-ray images of 65,240 patients for 14 thorax diseases.

Almost all current approaches for thorax disease detection seek to learn features for thorax diseases. They strive to exclude common anatomical features during the feature learning process and treat them as noise or outlier features. Because chest X-ray images are so rich in generic anatomical features, there’s a good chance that if a model tries to learn these features before attempting to learn the features for common thorax diseases, its performance can improve significantly.

## Method

Pre-processing the data, training the deep learning model, and visualising the results are the three important components of the proposed study in this research. The proposed work’s main goal is to determine the role of anatomical features in chest X-ray images for thorax disease classification based on the three components. Therefore, we’ll present a brief overview of the three components in this section.

### Dual-stream CNN network

Deep convolutional neural networks (DCNNs) have risen in popularity in recent years because of their substantial improvements in a range of applications, particularly image classification tasks. DCNN’s success can be attributed in large part to its deep architecture and end-to-end learning approach, which allows it to learn feature representations of the input. Furthermore, the primary goal of DCNN research is to create advanced networks and training algorithms to extract more discriminative features. There has been a lot of interest in increasing the capacity of DCNN, where the networks are generally built to be deeper or wider. In this research, we take a wider network approach and consider a DCNN to be made up of a network composed of the feature extractors of two subnetworks placed side by side, forming the Dual-stream network’s feature extractor. As a result, for an input image, two streams of features can be extracted, which are then combined to generate a unified representation of the final classifier for the overall classification.

**Figure 1:**
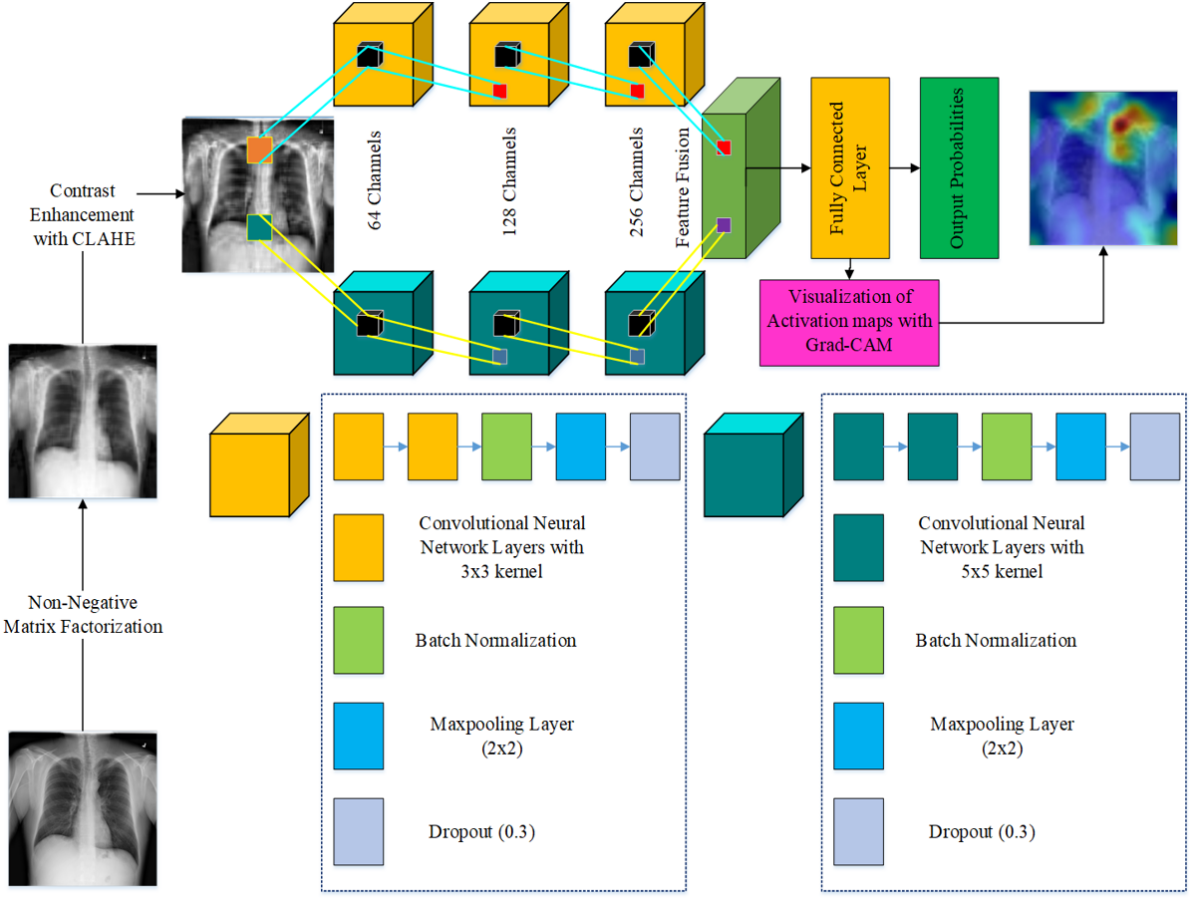
Architecture of the proposed model.

The ground truth is that each layer of CNNs learns filters of increasing complexity, similarly, the initial layers learn edges, corners, or in simple words boundaries between objects within an image. The middle layers learn features of parts of objects for instance when there is a face they might learn features for eyes or nose etc. Higher representations are found in the final layers, which learn to identify complete objects in various shapes and positions. Therefore, the model we use in our approach is a dual-stream network as shown in Fig. 1. As there are very few layers in both the streams they will mostly learn edge-like features, which are anatomical features in our case. The dual-stream technique allows the model to learn a large number of anatomical features, which are subsequently combined to form a feature bank.

The streams in proposed model are represented as *S*_1_ and *S*_2_. The streams *S*_1_ and *S*_2_ are based on three blocks each. The blocks contain two convolutional layers, a batch normalization layer and a dropout layer. The stream *S*_1_ uses 3 × 3 filters, whereas stream *S*_2_ uses 5 × 5 filters. The features learned by each stream *S*_1_ and *S*_2_ are formally represented as **F**_*S*_1__, and **F**_*S*_2__ respectively. The features **F**_*S*_1__ are generated using filter size 3 × 3, whereas **F**_*S*_2__ are the features generated by using filter size of 5 × 5. At each block, the streams *S*_1_ and *S*_2_ use a batch normalization and dropout layers. The streams also use a max-pooling layer with a filter size of 2 × 2 at each block. Finally the features **F**_*S*_1__ and **F**_*S*_2__ are concatenated into **F**_*total*_, as shown in Eq. (1).

(1) \begin{eqnarray*}{\mathbf{F}}_{total}= \left( \begin{array}{@{}c@{}} \displaystyle {\mathbf{F}}_{{S}_{1}}\\ \displaystyle {\mathbf{F}}_{{S}_{2}} \end{array} \right) \end{eqnarray*}

The features **F**_*total*_ are concatenation of the features **F**_*S*_1__ and **F**_*S*_2__. Such a concatenation of the features makes the model more robust on all the three classification problems of gender, age, and disease detection.

### Image pre-processing

To explore the anatomical features in chest X-ray images, the model is forced to learn anatomical features first and ignore the disease-related features as much as possible. To achieve this goal, the chest X-ray images are pre-processed with NMF and CLAHE to suppress the disease-related features. When the model is trained on the pre-processed images, it focuses more on anatomical features to detect gender and age, which are empirically presented in the experimental section.

#### Feature suppression with NMF

For feature supression, lets assume that there is a non-negative matrix Λ with *m* × *n* dimensions, NMF produces two non-negative matrices denoted as Ψ, and *H*. The matrix Ψ has *m* × *κ* (*κ* is often set by *κ* < *nm*/(*n* + *m*)) dimensions and *H* has *κ* × *n* dimensions. The two matrices minimize the following optimization function:

(2) \begin{eqnarray*}P=\min _{\Psi ,H\geqslant 0}{ \left\| \Lambda -\Psi H \right\| }_{2}\end{eqnarray*}

There is a basis vector (linearly independent) at each column of the matrix Ψ, and there are weights at each column of the matrix *H*, which estimate the respective columns in Λ by joining from Ψ that is a basis form. Hence the product Ψ*H* may be considered as a compressed version of the data in the matrix Λ. The objective function can be re-formalized as follows:

(3) \begin{eqnarray*}F=\sum _{i=1}^{m}\sum _{j=1}^{n}[{\Lambda }_{ij}\log \nolimits (\Psi H)_{ij}-(\Psi H)_{ij}].\end{eqnarray*}

**Figure 2:**
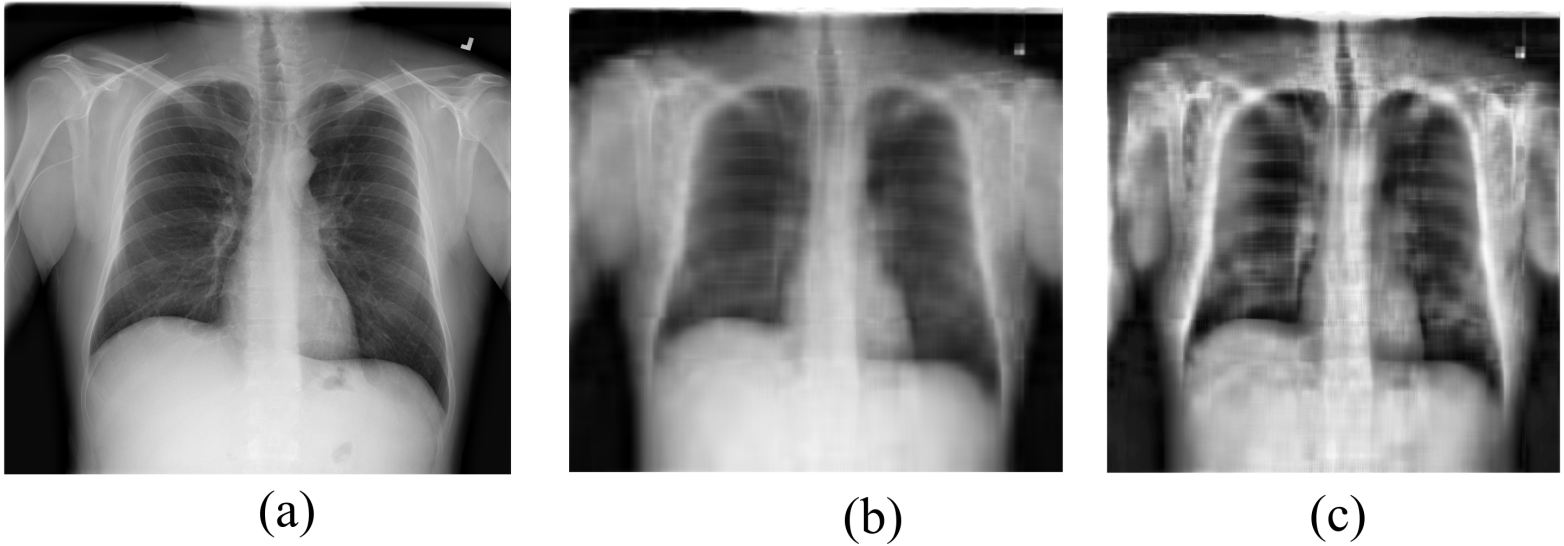
Pre-processing of image. (A) Original image, (B) image compressed with NMF, (C) image pre-processed with Contrast Limited Adaptive Histogram Equalization (CLAHE).

Figure 2B shows the effect of the NMF on chest X-ray images.

#### Contrast enhancement with CLAHE

One of the most popular methods of contrast enhancement for image classification in the recent past has been the Contrast Limited Adaptive Histogram Equalization(CLAHE) (Campos et al., 2019). Despite the recent emergence of many novel methods, CLAHE has been successfully applied in a variety of medical image processing applications, including cell image segmentation, breast ultrasound, and mammography image enhancement, retinal vascular image processing, and bone fracture image enhancement (El-bana, Al-Kabbany & Sharkas, 2020). The key benefit of utilizing CLAHE is that it is simpler and requires fewer processing resources without sacrificing performance. The core concept of CLAHE comprises the accomplishment of the histogram equalization by using interpolation to fix discrepancies between borders of non-overlapping sub-areas of the image. There are two important hyperparameters of CLAHE: the number of tiles (NT) and the clip limit (CL). The CL is a numerical quantity that regulates noise amplification. As soon as the histogram of each sub-area is computed, they are restructured in such a manner that its height does not go beyond a preferred “clip limit.” Then, the cumulative histogram is computed to accomplish the equalization. The NT is an integer quantity that governs the aggregate of non-overlapping sub-areas: based on its quantity, the image is distributed into many (typically squared) non-overlapping sections of identical dimensions. For instance, for a 512 × 512 image, the number of sections is normally chosen to be equivalent to 64 where NT = [8,8]. Figure 2C shows the effect of the CALHE on chest X-ray images.

### Margin loss

We adopt the margin loss (Xiao, Luo & Zhang, 2017) with dynamic weights to handle the class imbalance and unnecessarily instant over-fitting of the model. Initial experiments revealed that the margin loss performs better than the cross-entropy loss. Consequently, we decided to use the margin loss, as represented in Eq. (4).

(4) \begin{eqnarray*}{\Gamma }_{\kappa }={T}_{\kappa }max(0,{m}^{+}-\parallel {\nu }_{\kappa }{\parallel }^{2})\hspace*{10.00002pt}+\lambda (1-{T}_{\kappa })max(0,\parallel {\nu }_{\kappa }{\parallel }^{2}-{m}^{-})\end{eqnarray*}

In Eq. (4) *κ* = 1 if *κ* class is present, *m*^+^ = 0.9, *m*^−^ = 0.1 and *λ* = 0.5 is down-weighting. Here *ν*_*κ*_ represents the feature vector with *κ* number of classes. The model we’re employing can be utilized for multi-class classification as well as binary classification. Therefore *κ* can be any finite discrete number. The values of *m*^+^, *m*^−^ and *λ* dynamically handle the value of total loss Γ_*κ*_. Ultimately the loss is minimized gradually by controlling the imbalance among the *κ* classes.

### Visualizing the attention maps

The gradient-weighted class activation mapping (Grad-CAM) (Selvaraju, 2017) is used to highlight the discriminative regions and discover the learned features. The final convolutional layer yields *K* feature maps *A*^*k*^ ∈ *R*^*u*×*v*^ of size *u* × *v*. To obtain the Grad-CAM map, the gradient of score *y*^*c*^ regarding feature maps *A*^*k*^, that is, $ \frac{\partial {y}^{c}}{\partial {A}_{ij}^{k}} $, is streamed back, and global-average-pooled to acquire the weights:

(5) \begin{eqnarray*}{a}_{k}^{c}= \frac{1}{Z} \sum _{i}\sum _{j} \frac{\partial {y}^{c}}{\partial {A}_{ij}^{k}} \end{eqnarray*}

The Grad-CAM map ${L}_{Grad-CAM}^{c}\in {R}^{u\times v}$ is calculated as

(6) \begin{eqnarray*}{L}_{Grad-CAM}^{c}=ReLU({\Sigma }_{k}{a}_{k}c{A}^{k})\end{eqnarray*}

where *ReLU*(.) is the rectified linear unit, and *Z* is the number of pixels in the features map. By up-sampling ${L}_{Grad-CAM}^{c}$ to the size of the input image, the final Grad-CAM map is obtained.

### Training and validation process

Because training and validation are the most crucial aspects of any deep learning approach, we train and test the model accordingly. The proposed simple dual-stream model is trained in three stages. Stage I: The data pre-processing, includes the data augmentation and the image processing with NMF and CLAHE. Stage II: It is the main stage, where the classification of the chest X-ray images for gender, age, and disease detection is performed. Stage III: It is the visualization of the activation maps with *Gard-CAM*.

#### Stage I

The training data is augmented at this stage to even out the distribution of samples across classes. The augmentation is mostly based on a [ − 30, 30] degree horizontal rotation (Lv, Shao & Huang, 2017). The training dataset is divided into two versions after data augmentation. The first version is the original augmented version, while the second is the dataset with NMF and CLAHE pre-processing.

#### Stage II

This stage is based on training and testing of the model, hence, the model is then trained continuously based on the following three phases.

• Phase 1: The model is trained for gender detection on the pre-processed dataset with NMF and CLAHE.
• Phase 2: The model is trained again to detect age. The dataset used is also a processed dataset with NMF and CLAHE.
• Phase 3: The model trained in Phase 1 & 2 is trained further in this phase to detect thorax diseases. The dataset is not the pre-processed version, but the augmented version with original images.

#### Stage III

The model trained in every phase of *Stage II* is used to visualize the activation maps with *Grad-CAM* for identifying the chest zones under attention.

## Experiments

In this section, the training procedure, the evaluation of the trained model(s), and the visualization of the activation maps with *Grad-CAM* is presented.

### Dataset preparation

We use a public dataset chest X-ray14 provided by NIH (National Institutes of Health) (Wang et al., 2017). The dataset contains 112,116 labeled images for 14 thorax diseases together with a *no-finding* class. It contains images with single or multiple labels. The model is trained and tested in different versions for gender, age, and disease detection.

As there is a huge variance in the number of samples in the disease detection dataset, we apply a traditional data augmentation technique to balance the number of images in each class. Before applying the data augmentation, test images are chosen randomly for each class as follows:

• Gender detection: 400 images for each class
• Age detection: 400 images for each class
• Disease detection: 50 images for each class

The augmented dataset is then represented by two versions, Ver_ORG, and Ver_NMF.

• Ver_ORG: The original dataset (augmented).
• Ver_NMF: The augmented dataset pr-processed with NMF and CLAHE.

Moreover, each version of the dataset is divided into training and validation sets. 80% of each dataset for training and 10% for validation and 10% test is reserved.

### Training procedure

We train and test the model on a Windows 10 PC equipped with NVidia Gforce GTX 1060, having 16 GB of RAM, Intel Ci7 64 bit processor. All the simulations are performed on Keras with Tensorflow at the backend.

**Figure 3:**
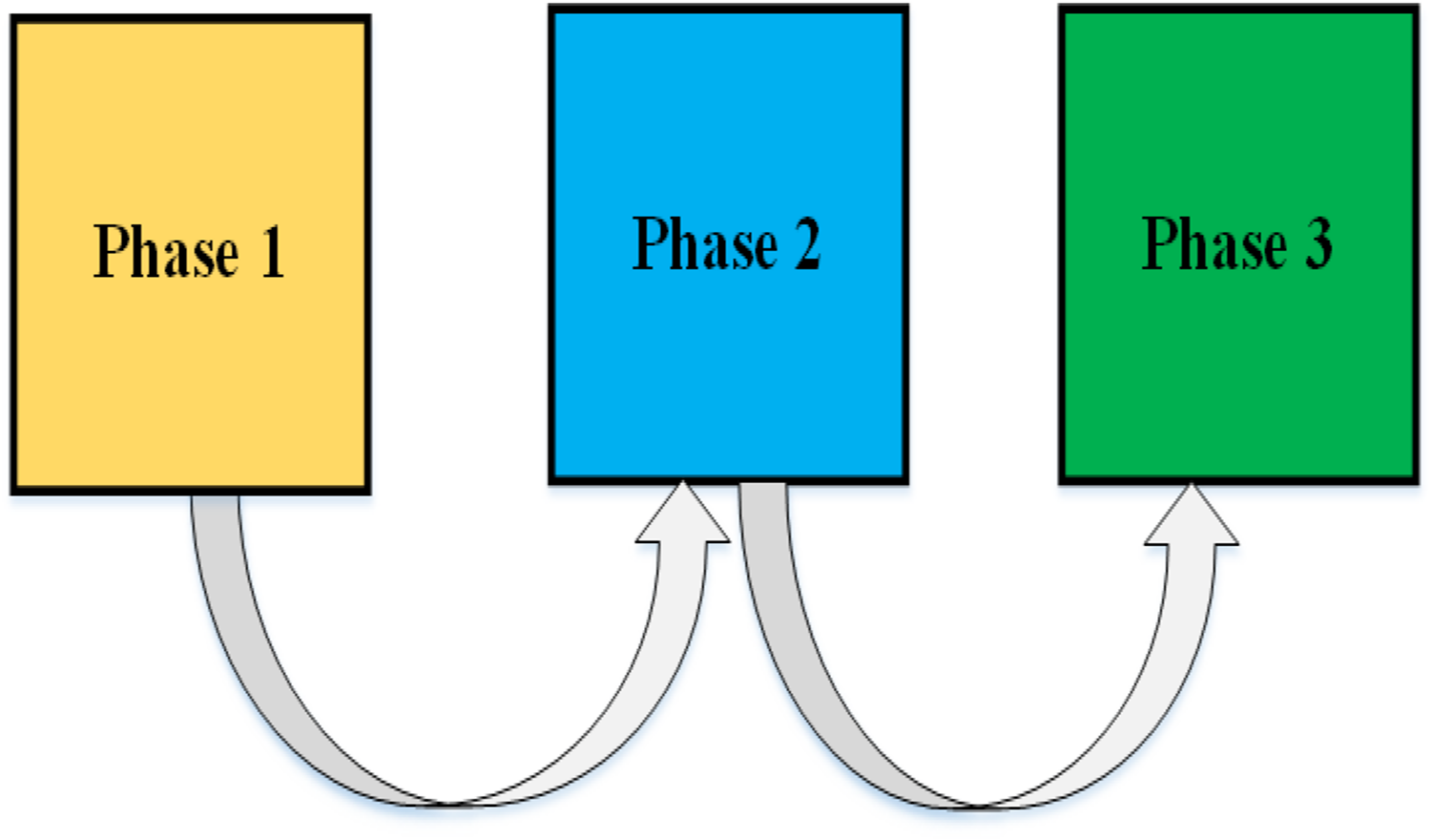
The model is trained continuously in three phases.

Since the model has different stages and phases for training, therefore abbreviations and descriptions of the model are given as shown in Table 1. There are 9 variants of the model (Table 1) each variant serves a unique purpose. The model is trained on two versions of the dataset that is Ver_ORG, and Ver_NMF. The main purpose of such variants is to investigate the performance of the model in different scenarios and determine the best scenario. The model is trained in three phases as depicted in Fig. 3.

**Table 1:** Short names for each version of the model. Note: the names are just symbolic.

Model	Description
GD_NMF	Gender detection model trained on dataset Ver NMF.
GD_NMF => AD_NMF	GD_NMF model trained again for age detection on dataset Ver NMF.
GD_NMF => AD_NMF => DD_ORG	Model GD_NMF => AD_NMF trained again for disease detection on dataset Ver ORG.
AD_NMF	Age detection model trained without correlative training on dataset Ver NMF.
GD_NMF => DD_ORG	GD_NMF model trained again for disease detection on dataset Ver ORG.
AD_NMF => DD_ORG	AD NMF model trained again for disease detection on dataset Ver ORG.
GD_ORG	Model trained for gender detection on dataset Ver ORG.
AD_ORG	Model trained for age detection on dataset Ver ORG.
DD_ORG	Model trained for disease detection on dataset Ver ORG.

**Figure 4:**
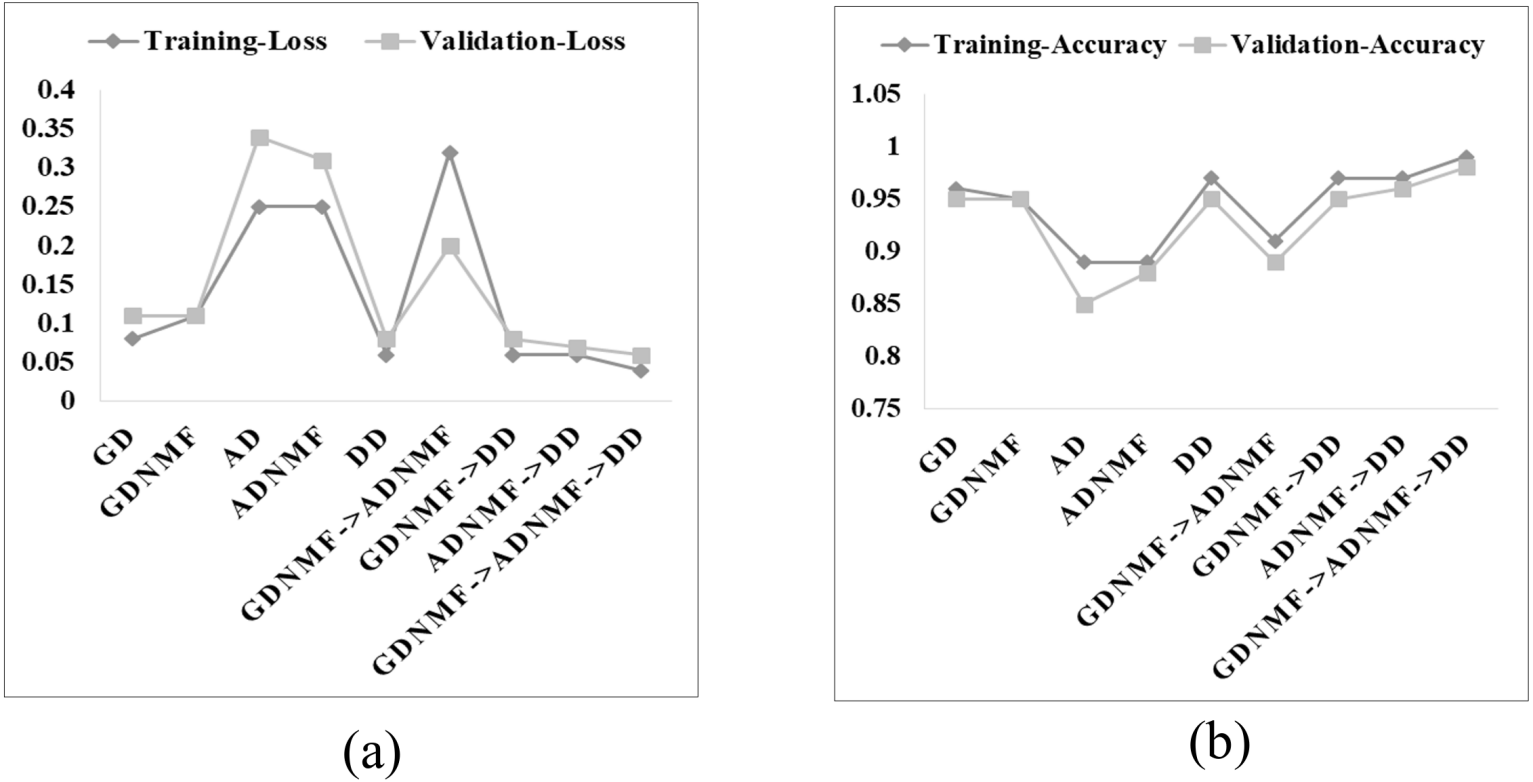
Final training accuracy, validation accuracy, training loss, and validation loss of the versions of the model (A–B).

In *Phase 1* of the training, the model is trained for gender detection with dataset Ver_NMF. Such a version of the model is named GD_NMF. Here the learning rate is kept as 0.001 and the number of epochs are 10 and the optimizer is Adam (). The training and validation accuracy along with loss for GD_NMF are encouraging as shown in Fig. 4.

In *Phase 2* of training, the model is trained to detect age with dataset Ver_NMF. The model is named as GD_NMF ⇒ AD_NMF, the model achieves high training and validation accuracy (Fig. 4). The learning rate, the number of epochs, and optimizer are the same as they were used for gender detection. In *Phase 3*, the model trained in *Phase 2* is trained further for disease detection on the dataset Ver_ORG. The model is named GD_NMF ⇒ AD_NMF ⇒DD_ORG. The reason for using the dataset Ver_ORG instead of Ver_NMF for disease detection is to use already learned features during the gender and age detection phase. Here the learning rate is 0.0001, the number of epochs is 100, and the optimizer is the same as it was used previously for gender and age detection. It can be observed that training and validation accuracy show a significant improvement for GD_NMF ⇒ AD_NMF ⇒DD_ORG (Fig. 4).

For comparison, the model is trained and named in different settings (Table 1). The corresponding training accuracy, validation accuracy, and losses are depicted in Fig. 4 for all the versions of the model.

### Evaluation

#### Evaluation metrics

For gender and age detection, the number of True Positives (TP), True Negatives (TN), False Positives (FP), and False Negatives (FN) were used to determine Accuracy, Precision, Recall, and F1 Scores (Juba & Le, 2019). We use the NIH Chest X-ray14 dataset according to usual protocol, which is to employ AUC (Area Under the Curve) as the major performance metric for thorax disease classification, as practically all of the literature suggests.

#### Results analysis

**Figure 5:**
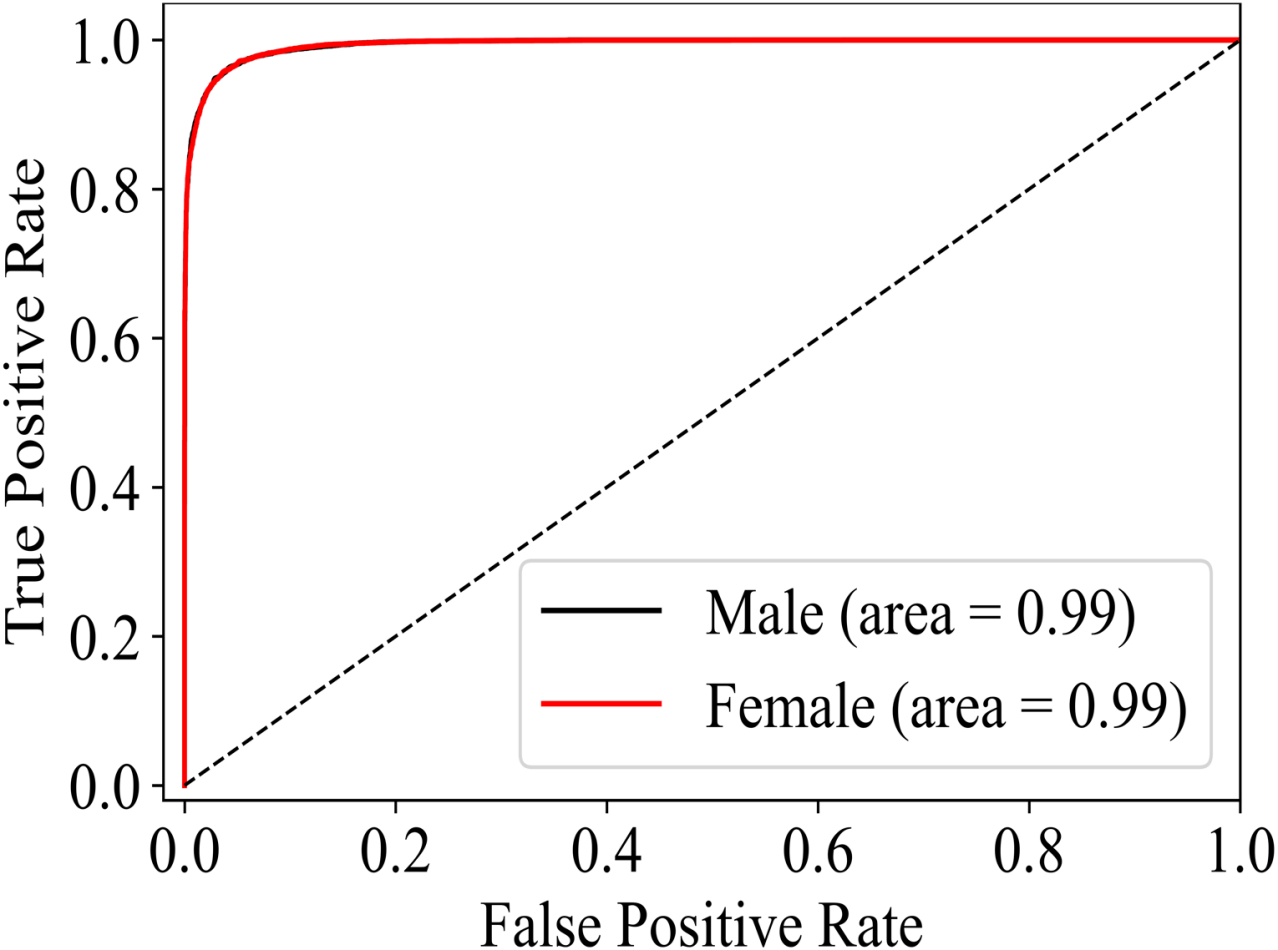
AUC for gender detection.

**Figure 6:**
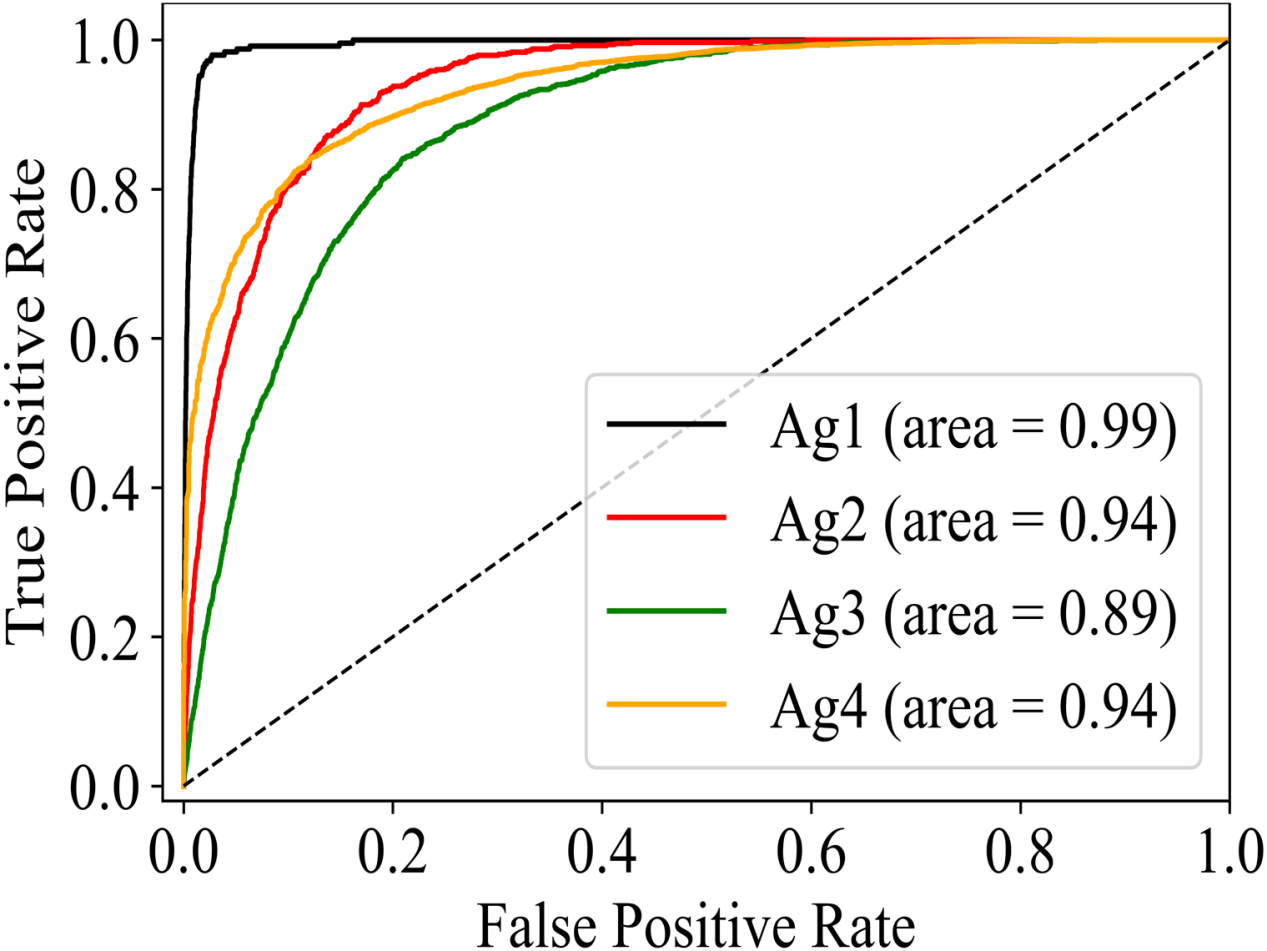
AUC for age detection.

**Figure 7:**
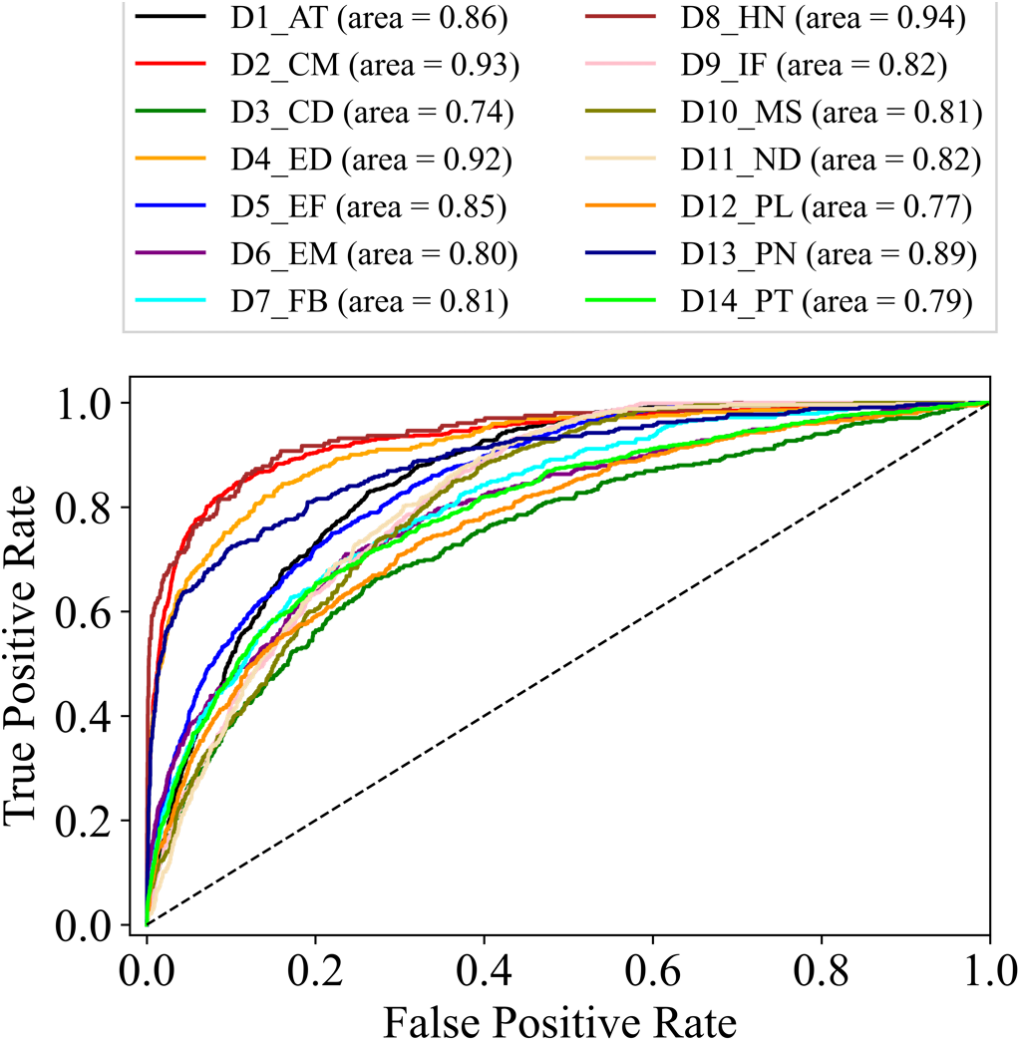
AUC for disease detection.

In the training phase, the training and validation accuracy are the indicators of the actual performance of the model. The best performing version of the model can easily be differentiated (Fig. 4) for gender, age, and disease detection even in the training phase. Furthermore, predictions on the test data on each version of the model are made to calculate the accuracy and F1 scores. For gender detection, the best performing version is GD_NMF. The accuracy (Table 2) is 0.96 for the class Male, and 0.94 for the class Female. For the age detection, the best accuracy for each of the class Age_1–10_, Aage_11–20_, Age_21–30_, and Age_31–120_ is 0.95, 0.87, 0.88, and 0.92 respectively. The different versions of the model (see Table 1) for disease detection are tested on the testing portion of the dataset. The disease detection part is analyzed only by using AUC values as most of the literature only reports AUC. The performances of each version of the model for disease detection are depicted in Table 3. It can be determined that GD_NMF ⇒ AD_NMF ⇒ DD_ORG performs the best. The respective AUC curves for gender, age and disease detection are depicted in Figs. 5, 6 and 7 respectively.

**Table 2:** Final results for gender, and age detection, F1: F1 Score, Note that the results of only the best performing version of the model for gender, and age detection are reported.

*N* = 400	TP	TN	FP	FN	Precision	Recall	Accuracy	F1	Average AUC
*Age*_1−10_	91	289	11	9	0.89	0.91	0.95	0.90	0.99
*Age*_11−20_	81	270	30	19	0.72	0.81	0.87	0.76	0.94
*Age*_21−30_	62	290	10	38	0.86	0.62	0.88	0.72	0.89
*Age*_31−120_	92	277	23	8	0.80	0.92	0.92	0.85	0.94
Male	98	47	3	2	0.97	0.98	0.96	0.97	0.99
Female	95	46	4	5	0.95	0.95	0.94	0.95	0.99

**Table 3:** Average AUC for disease detection.

Model	Average AUC
DD_ORG	0.800
GD_NMF ⇒ DD_ORG	0.809
AD_NMF ⇒ DD_ORG	0.810
GD_NMF ⇒ AD_NMF ⇒ DD_ORG	0.839
VGG-16	0.763
GoogleNet	0.780
ResNet-101	0.792
DenseNet-121	0.826

The analysis of the results in Table 3 shows that when the model is trained using the proposed technique it produces better results as compared to traditional methods where pre-trained models are used. It can be observed the version GD_NMF ⇒ AD_NMF ⇒ DD_ORG outperforms VGG-16, GoogleNet, ResNet-101, and DenseNet-121. The model produces an AUC of 0.839 which is better than the best performing pre-trained model DenseNet-121 having an AUC of 0.826. Other than disease detection the model has better accuracy for gender and age detection. By observing the results presented in Table 2, the model trained on data pre-processed with NMF and CLAHE has better performance in both the cases that are gender and age detection. The results indicate the efficacy of using the proposed technique compared with conventional methods.

**Figure 8:**
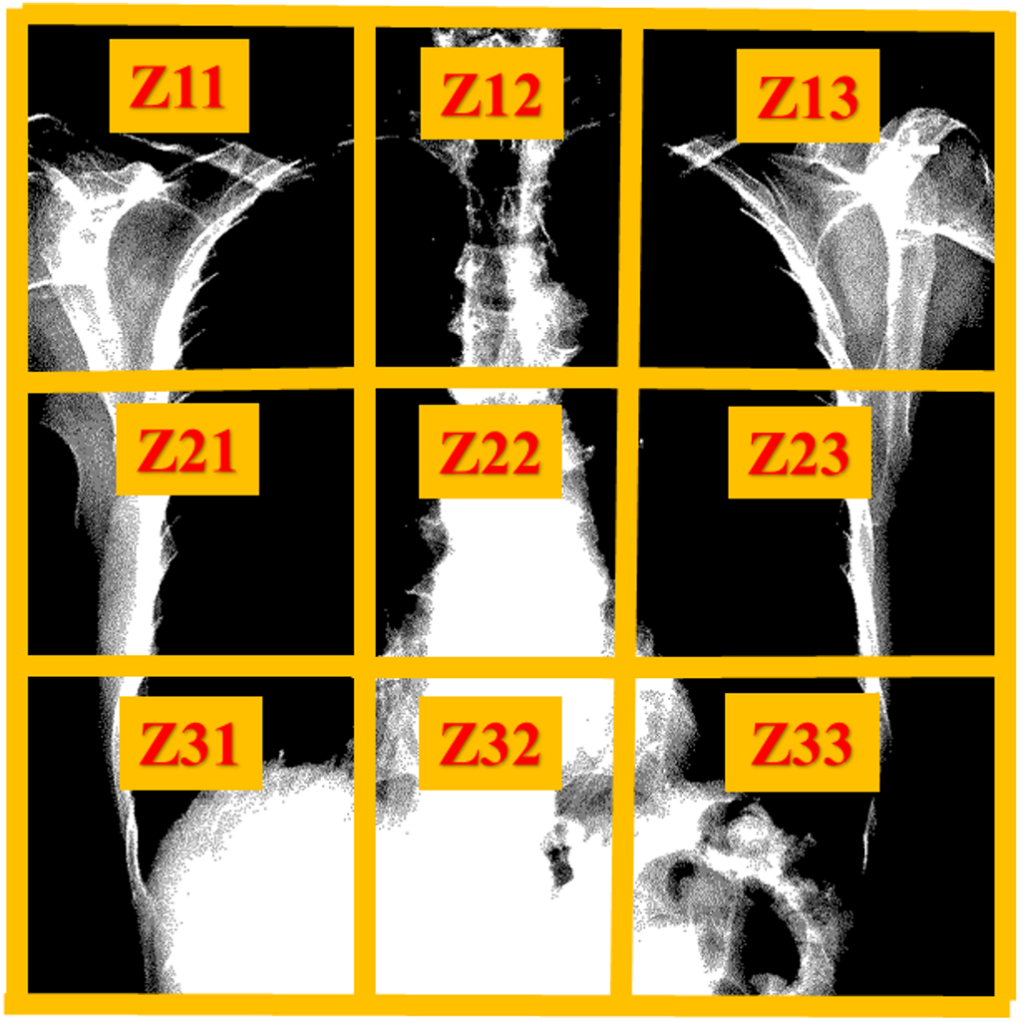
Zones of chest X-ray images for attention visualization.

**Figure 9:**
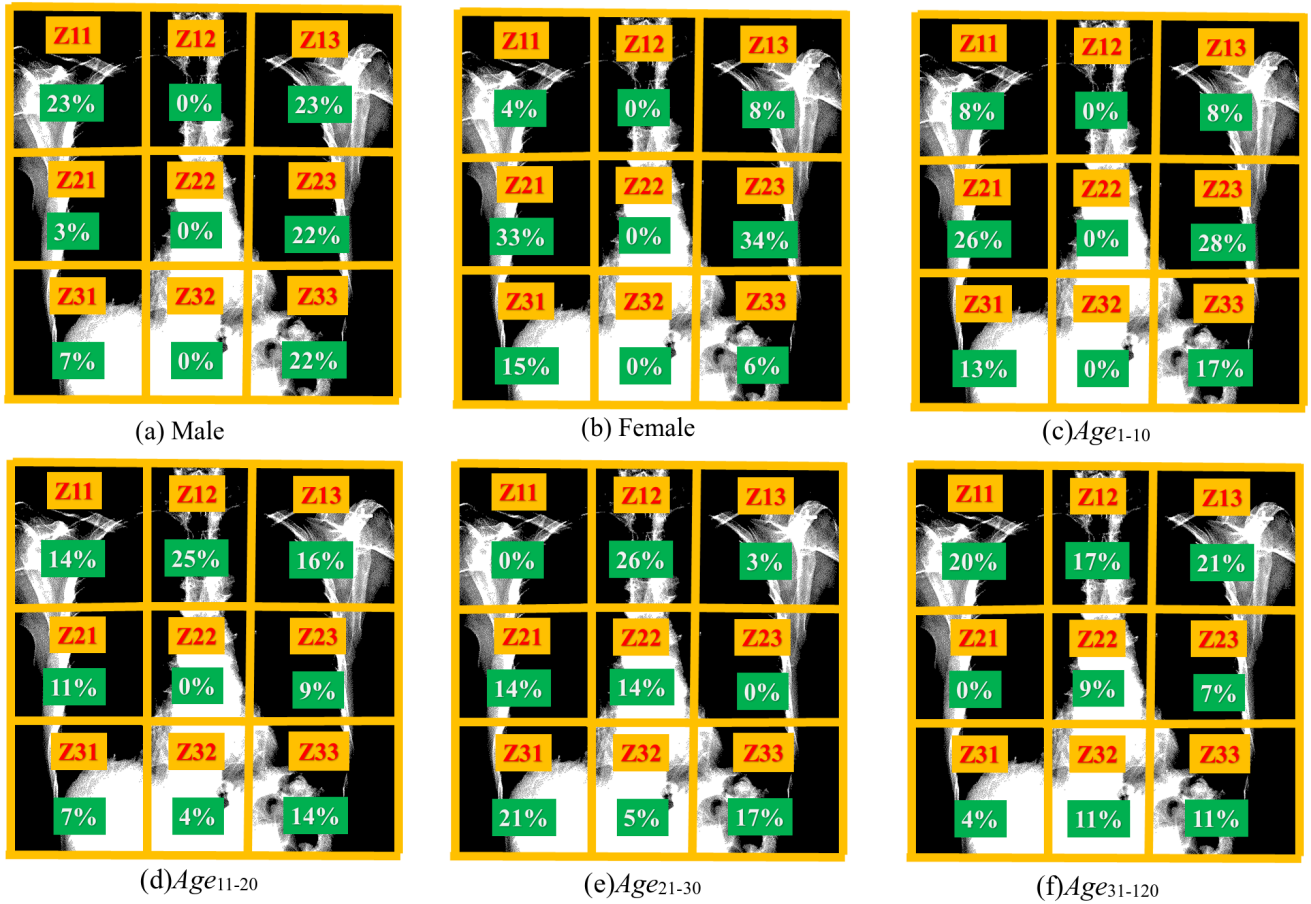
The graphs show the attention of the models in each zone to learn the features for the classes (A–F). The higher bars show that the model is focusing more on that particular zone to learn features. For instance, the gender detection model pays more attention to learn features for class Male at Z11 and Z13. Similarly, the model pays most attention at Z11 and Z13 to learn features for class Age_31–120_.

**Figure 10:**
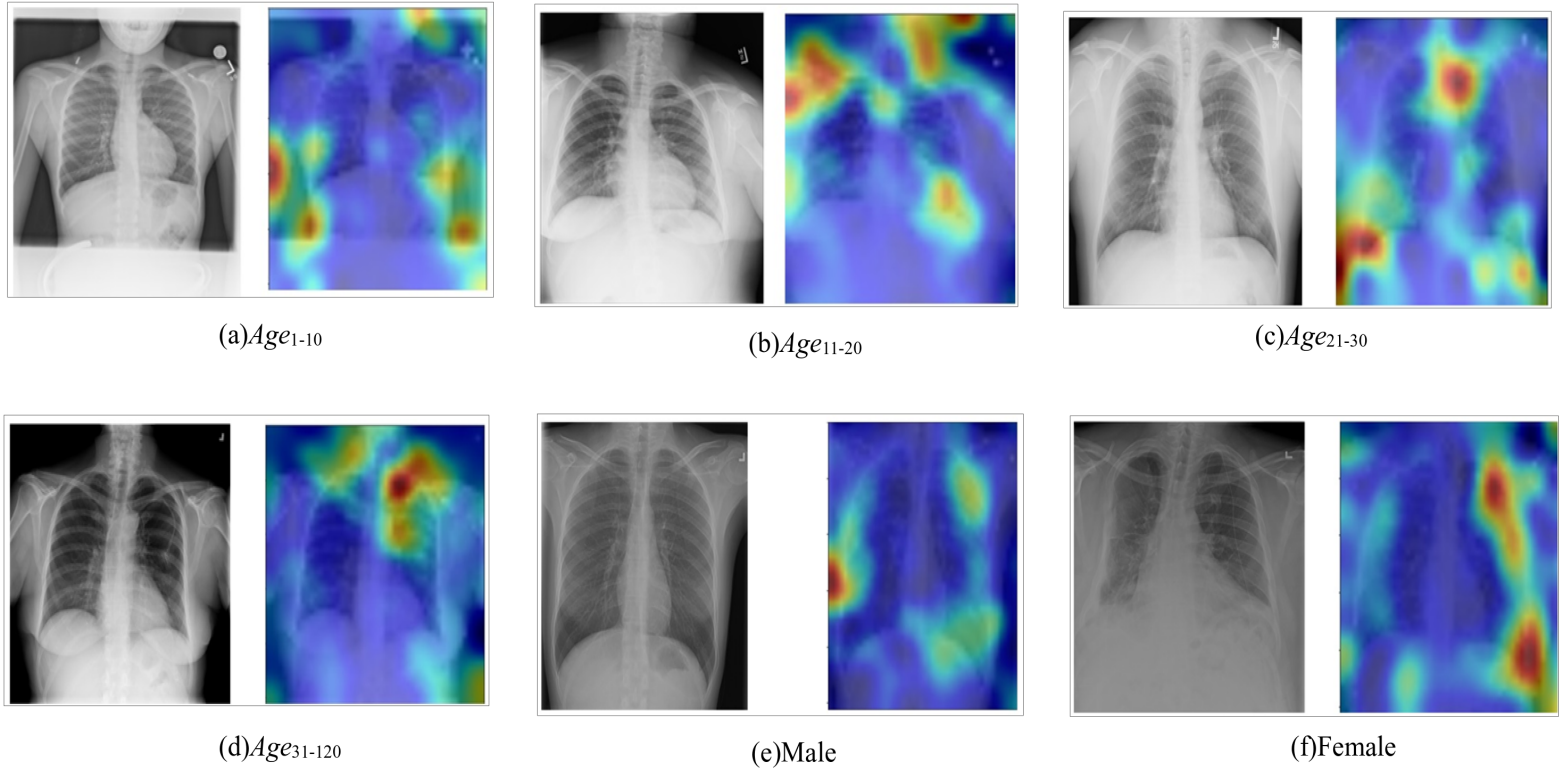
Visualization of the area of attention for gender and age detection using Grad-CAm (A–F).

To further validate that the model trained for gender and age on Ver_NMF improves disease detection performance, the area of attention for gender and age detection is visualized. The chest X-ray image is divided into 9 zones (Fig. 8). Specifically, Z11 and Z13 represent the shoulders region, Z12 represents spinal bones around the neck area. Z21 and Z23 represent the right and the left sides of the rib cage, respectively. Z22 and Z32 represent the bones along the spine in the range of the chest area. Z31 and Z33 represent the right bottom part and the left bottom part of the chest. Correctly classified test images are used to observe the area of attention on the best performing version(s) of the model. Figure 9 illustrates the zone-wise area of attention for the classes of gender and age, wheres Figure 10 shows some examples of the area of attention for gender and age detection using Grad-CAM. Using the reference lines as depicted in Fig. 8, the zone(s) under attention for each correctly classified sample can be noted. The main objective for zone-wise attention identification is to discover the area where the model is more focused on learning the features for each class.

**Figure 11:**
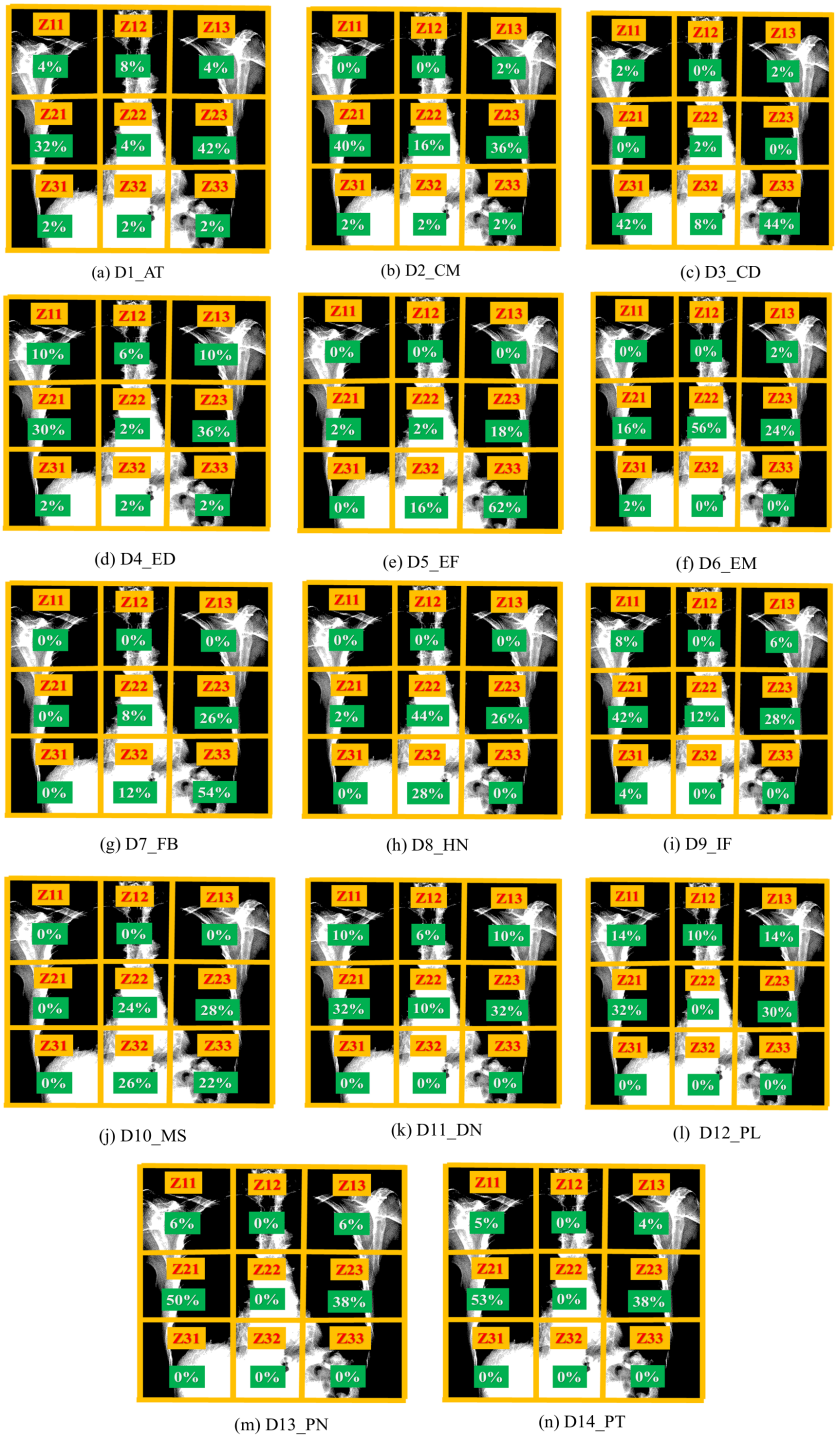
Graph depicting the attention of each disease class (A–N). The graph shows that almost all the classes get significant attention at Z21 and Z23. D1_AT:Atelectasis, D2_CM:Cardiomegaly, D3_CD:Consolidation, D4_ED:Edema, D5_EF:Effusion, D6_EM:Emphysema, D7_FB:Fibrosis, D8_HN:Hernia, D9_IF:Infiltration, D10_MS:Mass, D11_ND:Nodule, D12_PL:Plueral Thickening, D13_PN:Pneumonia, D14_PT:Pneumothorax.

For the gender detection, as shown in Fig. 9, the area of attention for male samples (Male) is mostly around Z11 and Z13. The zones Z12, Z22, and Z32 play no role for male samples. For detection of age, the attention is not as straightforward as compared to the gender detection model. For the class Age_1–10_, the attention is focused on Z21 and Z23. The zones Z12, Z22, and Z32 play no role in the detection of age. Z11, Z13, Z31, and Z33 have significant attention for class Age_1–10_. Class Age_11–20_ receives most of its attention around the upper side of the chest (Z11, Z12, Z13). Z22 plays no role for the class Age_11–20_.

The attention for the class Age_21–30_ is not quite clear, which is mostly scattered around the chest bones. The zones Z11 and Z23 play no role for class Age_21–30_. As shown in Fig. 9, class Age_31–120_ receives attention mostly around Z11, Z12, and Z13, which are at the upper part of the chest. The zone Z21 has no impact on age detection for class Age_31–120_. The rest of the zones (Z22, Z23, Z31, Z32, and Z33) play important roles for class Age_31–120_.

As shown in Fig. 11, the attention for disease detection is mostly around Z21 and Z23. The zones Z11 and Z13 get a little attention for almost all the diseases, in contrast, these zones receive high attention for gender and age. The visualization of the attention maps and the zone-wise investigation demonstrate that the model learns features from chest zones, where there is little chance of the presence of a thorax disease. Hence, the majority of the features of gender and age detection are the outliers for disease detection. The training of the model (used for gender and age detection) continuously for disease detection takes less training time and produces better performance.

### Comparison of the proposed model with state-of-the-art methods

As the objective of the proposed technique is not only to detect gender, age, and thorax diseases in chest X-ray images but also to find a relation between the features learned for one task to improve the performance of the other task. The results discussed in the preceding sub-sections show that the objective of the proposed method is greatly achieved by observing the performance of the model. Most of the state-of-the-art techniques analyze the performance based on AUC; therefore, Table 4 depicts the average AUC reported by state-of-the-art methods.

**Table 4:** Comparison of the results with state-of-the-art methods.

Author	Model/Method	Dataset	Average AUC	Year
Huang et al. (2020)	FHRNet	ChestX-ray14	0.812	2020
Chen et al. (2019)	Dualchexnet	ChestX-ray14	0.823	2019
Wang et al. (2020b)	DenseNet-121	ChestX-ray14	0.820	2020
Wang et al. (2020a)	DenseNet-121	ChestX-ray14	0.826	2020
Proposed method	Dual-Stream CNN	ChestX-ray14	0.857	2021

It can be observed that the proposed method outperforms the previous techniques. The comparisons of the results are based on the same data i-e ChestX-ray14. The main reason for the higher overall performance of the model may be attributed to the unique method of the usage of anatomical features previously discovered for gender and age detection. Those anatomical features had been mostly learned because of highlighted structural detail in the chest X-ray images because of efficiently pre-processed images with NMF and CLAHE. When the model already is aware of structural information in the chest rays it learns the features for thorax diseases more efficiently.

## Conclusion

The significance of gender and age detection features for thorax disease classification was examined and revealed in this paper. Empirical analysis demonstrates that learning anatomical features initially in the model’s learning process improves the model’s ability to learn localized features of thorax diseases. Similarly, the dual-stream technique improves the model’s ability to learn anatomical features, as does pre-processing chest X-ray images with NMF and CLAHE. When the explored anatomical knowledge is applied to thorax disease classification, the model gains a better initialization and, as a result, it not only generalizes better than traditional approaches but also performs much better. We demonstrated that the model performs better when trained for gender and age detection on images pre-processed with NMF and CLAHE. When a model is trained to identify gender and age detection, it typically learns anatomical features, which aid in the model’s ability to learn features for thorax disease classification. The model produces state-of-the-art results for gender and age detection and competitive results for thorax disease classification. We identified significant parts of chest X-ray images for gender, age, and disease detection by visualizing and analyzing the area of attention for each class.

## Supplemental Information

10.7717/peerj-cs.738/supp-1 Supplemental Information 1 Code and Dataset Labels

## Additional Information and Declarations

### Data Availability

The following information was supplied regarding data availability: The code is available at GitHub:
https://github.com/mumtazali3205/Features_Gender_Age_CNN. The dataset is available at Box-NIH:
https://nihcc.app.box.com/v/ChestXray-NIHCC.
